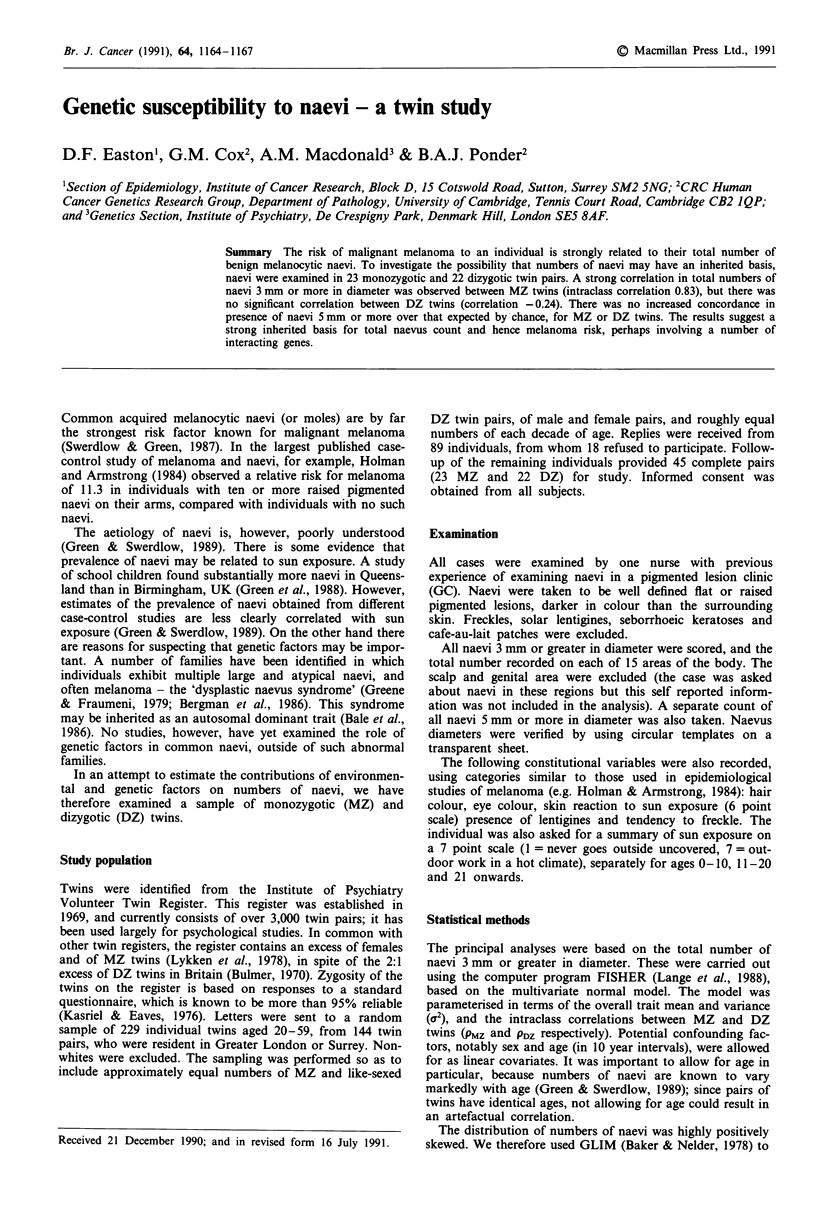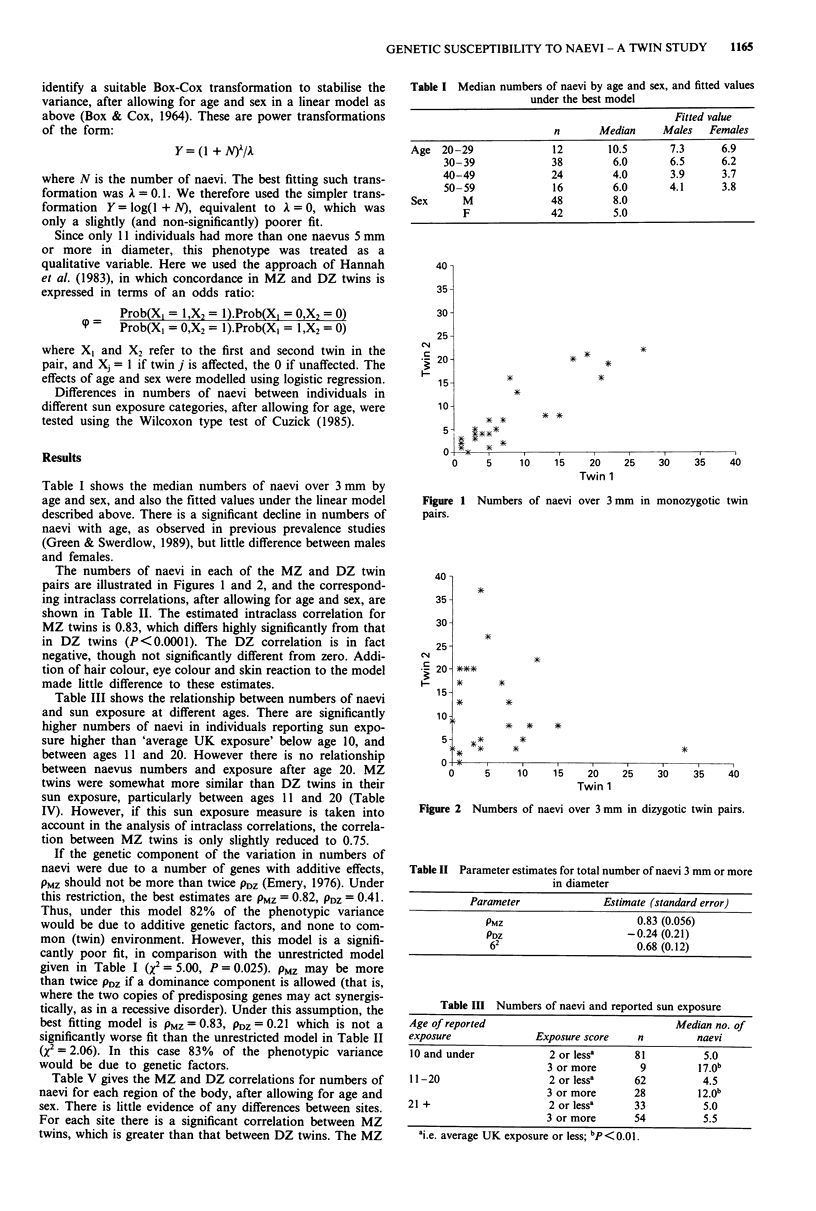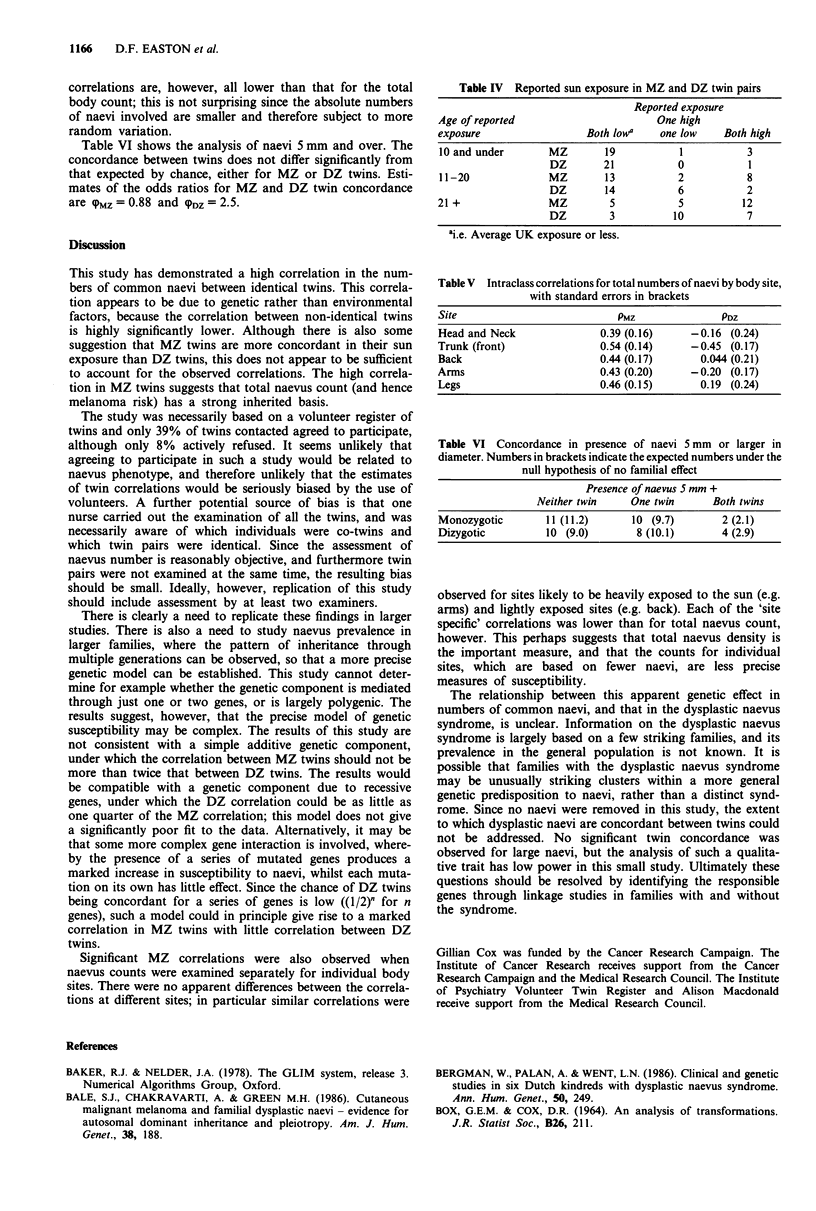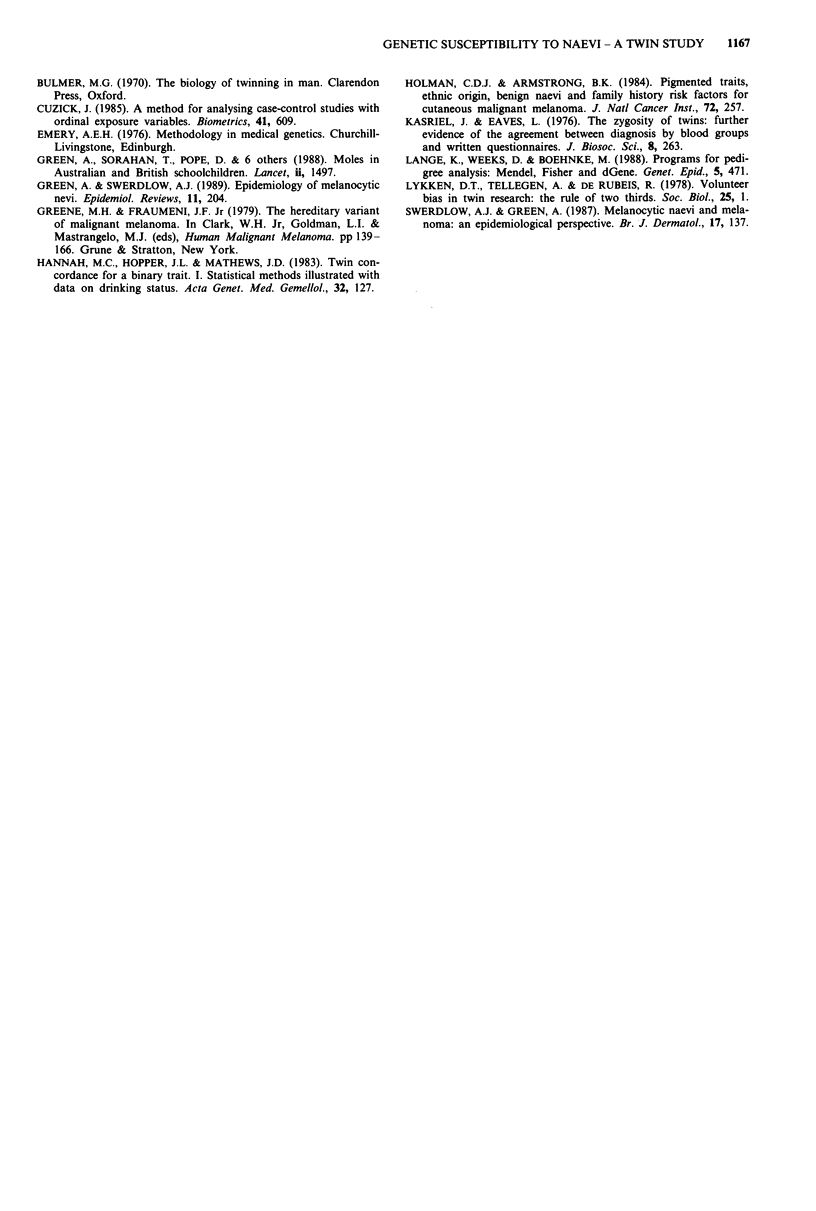# Genetic susceptibility to naevi--a twin study.

**DOI:** 10.1038/bjc.1991.483

**Published:** 1991-12

**Authors:** D. F. Easton, G. M. Cox, A. M. Macdonald, B. A. Ponder

**Affiliations:** Section of Epidemiology, Institute of Cancer Research, Sutton, Surrey.

## Abstract

The risk of malignant melanoma to an individual is strongly related to their total number of benign melanocytic naevi. To investigate the possibility that numbers of naevi may have an inherited basis, naevi were examined in 23 monozygotic and 22 dizygotic twin pairs. A strong correlation in total numbers of naevi 3 mm or more in diameter was observed between MZ twins (intraclass correlation 0.83), but there was no significant correlation between DZ twins (correlation -0.24). There was no increased concordance in presence of naevi 5 mm or more over that expected by chance, for MZ or DZ twins. The results suggest a strong inherited basis for total naevus count and hence melanoma risk, perhaps involving a number of interacting genes.


Br. J. Cancer (1991), 64, 1164-1167 © Macmillan Press Ltd., 1991

### Genetic susceptibility to naevi – a twin study

D.F. Easton[1], G.M. Cox[2], A.M. Macdonald[3] & B.A.J. Ponder[2]

[1]*Section of Epidemiology, Institute of Cancer Research, Block D, 15 Cotswold Road, Sutton, Surrey SM2 5NG;* [2]*CRC Human Cancer Genetics Research Group, Department of Pathology, University of Cambridge, Tennis Court Road, Cambridge CB2 1QP; and* [3]*Genetics Section, Institute of Psychiatry, De Crespigny Park, Denmark Hill, London SE5 8AF.*

**Summary** The risk of malignant melanoma to an individual is strongly related to their total number of benign melanocytic naevi. To investigate the possibility that numbers of naevi may have an inherited basis, naevi were examined in 23 monozygotic and 22 dizygotic twin pairs. A strong correlation in total numbers of naevi 3 mm or more in diameter was observed between MZ twins (intraclass correlation 0.83), but there was no significant correlation between DZ twins (correlation −0.24). There was no increased concordance in presence of naevi 5 mm or more over that expected by chance, for MZ or DZ twins. The results suggest a strong inherited basis for total naevus count and hence melanoma risk, perhaps involving a number of interacting genes.

Common acquired melanocytic naevi (or moles) are by far the strongest risk factor known for malignant melanoma (Swerdlow & Green, 1987). In the largest published case-control study of melanoma and naevi, for example, Holman and Armstrong (1984) observed a relative risk for melanoma of 11.3 in individuals with ten or more raised pigmented naevi on their arms, compared with individuals with no such naevi.

The aetiology of naevi is, however, poorly understood (Green & Swerdlow, 1989). There is some evidence that prevalence of naevi may be related to sun exposure. A study of school children found substantially more naevi in Queensland than in Birmingham, UK (Green *et al.*, 1988). However, estimates of the prevalence of naevi obtained from different case-control studies are less clearly correlated with sun exposure (Green & Swerdlow, 1989). On the other hand there are reasons for suspecting that genetic factors may be important. A number of families have been identified in which individuals exhibit multiple large and atypical naevi, and often melanoma – the 'dysplastic naevus syndrome' (Greene & Fraumeni, 1979; Bergman *et al.*, 1986). This syndrome may be inherited as an autosomal dominant trait (Bale *et al.*, 1986). No studies, however, have yet examined the role of genetic factors in common naevi, outside of such abnormal families.

In an attempt to estimate the contributions of environmental and genetic factors on numbers of naevi, we have therefore examined a sample of monozygotic (MZ) and dizygotic (DZ) twins.

### Study population

Twins were identified from the Institute of Psychiatry Volunteer Twin Register. This register was established in 1969, and currently consists of over 3,000 twin pairs; it has been used largely for psychological studies. In common with other twin registers, the register contains an excess of females and of MZ twins (Lykken *et al.*, 1978), in spite of the 2:1 excess of DZ twins in Britain (Bulmer, 1970). Zygosity of the twins on the register is based on responses to a standard questionnaire, which is known to be more than 95% reliable (Kasriel & Eaves, 1976). Letters were sent to a random sample of 229 individual twins aged 20–59, from 144 twin pairs, who were resident in Greater London or Surrey. Non-whites were excluded. The sampling was performed so as to include approximately equal numbers of MZ and like-sexed DZ twin pairs, of male and female pairs, and roughly equal numbers of each decade of age. Replies were received from 89 individuals, from whom 18 refused to participate. Follow-up of the remaining individuals provided 45 complete pairs (23 MZ and 22 DZ) for study. Informed consent was obtained from all subjects.

### Examination

All cases were examined by one nurse with previous experience of examining naevi in a pigmented lesion clinic (GC). Naevi were taken to be well defined flat or raised pigmented lesions, darker in colour than the surrounding skin. Freckles, solar lentigines, seborrhoeic keratoses and cafe-au-lait patches were excluded.

All naevi 3 mm or greater in diameter were scored, and the total number recorded on each of 15 areas of the body. The scalp and genital area were excluded (the case was asked about naevi in these regions but this self reported information was not included in the analysis). A separate count of all naevi 5 mm or more in diameter was also taken. Naevus diameters were verified by using circular templates on a transparent sheet.

The following constitutional variables were also recorded, using categories similar to those used in epidemiological studies of melanoma (e.g. Holman & Armstrong, 1984): hair colour, eye colour, skin reaction to sun exposure (6 point scale) presence of lentigines and tendency to freckle. The individual was also asked for a summary of sun exposure on a 7 point scale (1 = never goes outside uncovered, 7 = outdoor work in a hot climate), separately for ages 0–10, 11–20 and 21 onwards.

### Statistical methods

The principal analyses were based on the total number of naevi 3 mm or greater in diameter. These were carried out using the computer program FISHER (Lange *et al.*, 1988), based on the multivariate normal model. The model was parameterised in terms of the overall trait mean and variance ($\sigma^2$), and the intraclass correlations between MZ and DZ twins ($\rho_{MZ}$ and $\rho_{DZ}$ respectively). Potential confounding factors, notably sex and age (in 10 year intervals), were allowed for as linear covariates. It was important to allow for age in particular, because numbers of naevi are known to vary markedly with age (Green & Swerdlow, 1989); since pairs of twins have identical ages, not allowing for age could result in an artefactual correlation.

The distribution of numbers of naevi was highly positively skewed. We therefore used GLIM (Baker & Nelder, 1978) to

Received 21 December 1990; and in revised form 16 July 1991.

identify a suitable Box-Cox transformation to stabilise the variance, after allowing for age and sex in a linear model as above (Box & Cox, 1964). These are power transformations of the form:

$$Y = (1 + N)^{\lambda}/\lambda$$

where $N$ is the number of naevi. The best fitting such transformation was $\lambda = 0.1$. We therefore used the simpler transformation $Y = \log(1 + N)$, equivalent to $\lambda = 0$, which was only a slightly (and non-significantly) poorer fit.

Since only 11 individuals had more than one naevus 5 mm or more in diameter, this phenotype was treated as a qualitative variable. Here we used the approach of Hannah *et al.* (1983), in which concordance in MZ and DZ twins is expressed in terms of an odds ratio:

$$\varphi = \frac{\text{Prob}(X_1 = 1, X_2 = 1).\text{Prob}(X_1 = 0, X_2 = 0)}{\text{Prob}(X_1 = 0, X_2 = 1).\text{Prob}(X_1 = 1, X_2 = 0)}$$

where $X_1$ and $X_2$ refer to the first and second twin in the pair, and $X_j = 1$ if twin $j$ is affected, the 0 if unaffected. The effects of age and sex were modelled using logistic regression.

Differences in numbers of naevi between individuals in different sun exposure categories, after allowing for age, were tested using the Wilcoxon type test of Cuzick (1985).

### Results

Table I shows the median numbers of naevi over 3 mm by age and sex, and also the fitted values under the linear model described above. There is a significant decline in numbers of naevi with age, as observed in previous prevalence studies (Green & Swerdlow, 1989), but little difference between males and females.

The numbers of naevi in each of the MZ and DZ twin pairs are illustrated in Figures 1 and 2, and the corresponding intraclass correlations, after allowing for age and sex, are shown in Table II. The estimated intraclass correlation for MZ twins is 0.83, which differs highly significantly from that in DZ twins ($P < 0.0001$). The DZ correlation is in fact negative, though not significantly different from zero. Addition of hair colour, eye colour and skin reaction to the model made little difference to these estimates.

Table III shows the relationship between numbers of naevi and sun exposure at different ages. There are significantly higher numbers of naevi in individuals reporting sun exposure higher than 'average UK exposure' below age 10, and between ages 11 and 20. However there is no relationship between naevus numbers and exposure after age 20. MZ twins were somewhat more similar than DZ twins in their sun exposure, particularly between ages 11 and 20 (Table IV). However, if this sun exposure measure is taken into account in the analysis of intraclass correlations, the correlation between MZ twins is only slightly reduced to 0.75.

If the genetic component of the variation in numbers of naevi were due to a number of genes with additive effects, $\rho_{MZ}$ should not be more than twice $\rho_{DZ}$ (Emery, 1976). Under this restriction, the best estimates are $\rho_{MZ} = 0.82$, $\rho_{DZ} = 0.41$. Thus, under this model 82% of the phenotypic variance would be due to additive genetic factors, and none to common (twin) environment. However, this model is a significantly poor fit, in comparison with the unrestricted model given in Table I ($\chi^2 = 5.00$, $P = 0.025$). $\rho_{MZ}$ may be more than twice $\rho_{DZ}$ if a dominance component is allowed (that is, where the two copies of predisposing genes may act synergistically, as in a recessive disorder). Under this assumption, the best fitting model is $\rho_{MZ} = 0.83$, $\rho_{DZ} = 0.21$ which is not a significantly worse fit than the unrestricted model in Table II ($\chi^2 = 2.06$). In this case 83% of the phenotypic variance would be due to genetic factors.

Table V gives the MZ and DZ correlations for numbers of naevi for each region of the body, after allowing for age and sex. There is little evidence of any differences between sites. For each site there is a significant correlation between MZ twins, which is greater than that between DZ twins. The MZ

**Table I** Median numbers of naevi by age and sex, and fitted values under the best model

| | | | | Fitted value | |
|---|---|---|---|---|---|
| | | *n* | *Median* | *Males* | *Females* |
| Age | 20–29 | 12 | 10.5 | 7.3 | 6.9 |
| | 30–39 | 38 | 6.0 | 6.5 | 6.2 |
| | 40–49 | 24 | 4.0 | 3.9 | 3.7 |
| | 50–59 | 16 | 6.0 | 4.1 | 3.8 |
| Sex | M | 48 | 8.0 | | |
| | F | 42 | 5.0 | | |

**Figure 1** Numbers of naevi over 3 mm in monozygotic twin pairs.

**Figure 2** Numbers of naevi over 3 mm in dizygotic twin pairs.

**Table II** Parameter estimates for total number of naevi 3 mm or more in diameter

| *Parameter* | *Estimate (standard error)* |
|---|---|
| $\rho_{MZ}$ | 0.83 (0.056) |
| $\rho_{DZ}$ | −0.24 (0.21) |
| $\sigma^2$ | 0.68 (0.12) |

**Table III** Numbers of naevi and reported sun exposure

| *Age of reported exposure* | *Exposure score* | *n* | *Median no. of naevi* |
|---|---|---|---|
| 10 and under | 2 or less[a] | 81 | 5.0 |
| | 3 or more | 9 | 17.0[b] |
| 11–20 | 2 or less[a] | 62 | 4.5 |
| | 3 or more | 28 | 12.0[b] |
| 21 + | 2 or less[a] | 33 | 5.0 |
| | 3 or more | 54 | 5.5 |

[a]i.e. average UK exposure or less; [b]$P < 0.01$.

correlations are, however, all lower than that for the total body count; this is not surprising since the absolute numbers of naevi involved are smaller and therefore subject to more random variation.

Table VI shows the analysis of naevi 5 mm and over. The concordance between twins does not differ significantly from that expected by chance, either for MZ or DZ twins. Estimates of the odds ratios for MZ and DZ twin concordance are $\varphi_{MZ} = 0.88$ and $\varphi_{DZ} = 2.5$.

**Table IV** Reported sun exposure in MZ and DZ twin pairs

| *Age of reported exposure* | | *Reported exposure* Both low[a] | One high one low | Both high |
|---|---|---|---|---|
| 10 and under | MZ | 19 | 1 | 3 |
| | DZ | 21 | 0 | 1 |
| 11–20 | MZ | 13 | 2 | 8 |
| | DZ | 14 | 6 | 2 |
| 21 + | MZ | 5 | 5 | 12 |
| | DZ | 3 | 10 | 7 |

[a]i.e. Average UK exposure or less.

**Table V** Intraclass correlations for total numbers of naevi by body site, with standard errors in brackets

| *Site* | $\rho_{MZ}$ | $\rho_{DZ}$ |
|---|---|---|
| Head and Neck | 0.39 (0.16) | −0.16 (0.24) |
| Trunk (front) | 0.54 (0.14) | −0.45 (0.17) |
| Back | 0.44 (0.17) | 0.044 (0.21) |
| Arms | 0.43 (0.20) | −0.20 (0.17) |
| Legs | 0.46 (0.15) | 0.19 (0.24) |

**Table VI** Concordance in presence of naevi 5 mm or larger in diameter. Numbers in brackets indicate the expected numbers under the null hypothesis of no familial effect

| | *Presence of naevus 5 mm +* Neither twin | One twin | Both twins |
|---|---|---|---|
| Monozygotic | 11 (11.2) | 10 (9.7) | 2 (2.1) |
| Dizygotic | 10 (9.0) | 8 (10.1) | 4 (2.9) |

### Discussion

This study has demonstrated a high correlation in the numbers of common naevi between identical twins. This correlation appears to be due to genetic rather than environmental factors, because the correlation between non-identical twins is highly significantly lower. Although there is also some suggestion that MZ twins are more concordant in their sun exposure than DZ twins, this does not appear to be sufficient to account for the observed correlations. The high correlation in MZ twins suggests that total naevus count (and hence melanoma risk) has a strong inherited basis.

The study was necessarily based on a volunteer register of twins and only 39% of twins contacted agreed to participate, although only 8% actively refused. It seems unlikely that agreeing to participate in such a study would be related to naevus phenotype, and therefore unlikely that the estimates of twin correlations would be seriously biased by the use of volunteers. A further potential source of bias is that one nurse carried out the examination of all the twins, and was necessarily aware of which individuals were co-twins and which twin pairs were identical. Since the assessment of naevus number is reasonably objective, and furthermore twin pairs were not examined at the same time, the resulting bias should be small. Ideally, however, replication of this study should include assessment by at least two examiners.

There is clearly a need to replicate these findings in larger studies. There is also a need to study naevus prevalence in larger families, where the pattern of inheritance through multiple generations can be observed, so that a more precise genetic model can be established. This study cannot determine for example whether the genetic component is mediated through just one or two genes, or is largely polygenic. The results suggest, however, that the precise model of genetic susceptibility may be complex. The results of this study are not consistent with a simple additive genetic component, under which the correlation between MZ twins should not be more than twice that between DZ twins. The results would be compatible with a genetic component due to recessive genes, under which the DZ correlation could be as little as one quarter of the MZ correlation; this model does not give a significantly poor fit to the data. Alternatively, it may be that some more complex gene interaction is involved, whereby the presence of a series of mutated genes produces a marked increase in susceptibility to naevi, whilst each mutation on its own has little effect. Since the chance of DZ twins being concordant for a series of genes is low ($(1/2)^n$ for $n$ genes), such a model could in principle give rise to a marked correlation in MZ twins with little correlation between DZ twins.

Significant MZ correlations were also observed when naevus counts were examined separately for individual body sites. There were no apparent differences between the correlations at different sites; in particular similar correlations were observed for sites likely to be heavily exposed to the sun (e.g. arms) and lightly exposed sites (e.g. back). Each of the 'site specific' correlations was lower than for total naevus count, however. This perhaps suggests that total naevus density is the important measure, and that the counts for individual sites, which are based on fewer naevi, are less precise measures of susceptibility.

The relationship between this apparent genetic effect in numbers of common naevi, and that in the dysplastic naevus syndrome, is unclear. Information on the dysplastic naevus syndrome is largely based on a few striking families, and its prevalence in the general population is not known. It is possible that families with the dysplastic naevus syndrome may be unusually striking clusters within a more general genetic predisposition to naevi, rather than a distinct syndrome. Since no naevi were removed in this study, the extent to which dysplastic naevi are concordant between twins could not be addressed. No significant twin concordance was observed for large naevi, but the analysis of such a qualitative trait has low power in this small study. Ultimately these questions should be resolved by identifying the responsible genes through linkage studies in families with and without the syndrome.

Gillian Cox was funded by the Cancer Research Campaign. The Institute of Cancer Research receives support from the Cancer Research Campaign and the Medical Research Council. The Institute of Psychiatry Volunteer Twin Register and Alison Macdonald receive support from the Medical Research Council.

### References

BAKER, R.J. & NELDER, J.A. (1978). The GLIM system, release 3. Numerical Algorithms Group, Oxford.

BALE, S.J., CHAKRAVARTI, A. & GREEN M.H. (1986). Cutaneous malignant melanoma and familial dysplastic naevi – evidence for autosomal dominant inheritance and pleiotropy. *Am. J. Hum. Genet.*, **38**, 188.

BERGMAN, W., PALAN, A. & WENT, L.N. (1986). Clinical and genetic studies in six Dutch kindreds with dysplastic naevus syndrome. *Ann. Hum. Genet.*, **50**, 249.

BOX, G.E.M. & COX, D.R. (1964). An analysis of transformations. *J.R. Statist Soc.*, **B26**, 211.

BULMER, M.G. (1970). The biology of twinning in man. Clarendon Press, Oxford.

CUZICK, J. (1985). A method for analysing case-control studies with ordinal exposure variables. *Biometrics*, **41**, 609.

EMERY, A.E.H. (1976). Methodology in medical genetics. Churchill-Livingstone, Edinburgh.

GREEN, A., SORAHAN, T., POPE, D. & 6 others (1988). Moles in Australian and British schoolchildren. *Lancet*, **ii**, 1497.

GREEN, A. & SWERDLOW, A.J. (1989). Epidemiology of melanocytic nevi. *Epidemiol. Reviews*, **11**, 204.

GREENE, M.H. & FRAUMENI, J.F. Jr (1979). The hereditary variant of malignant melanoma. In Clark, W.H. Jr, Goldman, L.I. & Mastrangelo, M.J. (eds), *Human Malignant Melanoma*. pp 139–166. Grune & Stratton, New York.

HANNAH, M.C., HOPPER, J.L. & MATHEWS, J.D. (1983). Twin concordance for a binary trait. I. Statistical methods illustrated with data on drinking status. *Acta Genet. Med. Gemellol.*, **32**, 127.

HOLMAN, C.D.J. & ARMSTRONG, B.K. (1984). Pigmented traits, ethnic origin, benign naevi and family history risk factors for cutaneous malignant melanoma. *J. Natl Cancer Inst.*, **72**, 257.

KASRIEL, J. & EAVES, L. (1976). The zygosity of twins: further evidence of the agreement between diagnosis by blood groups and written questionnaires. *J. Biosoc. Sci.*, **8**, 263.

LANGE, K., WEEKS, D. & BOEHNKE, M. (1988). Programs for pedigree analysis: Mendel, Fisher and dGene. *Genet. Epid.*, **5**, 471.

LYKKEN, D.T., TELLEGEN, A. & DE RUBEIS, R. (1978). Volunteer bias in twin research: the rule of two thirds. *Soc. Biol.*, **25**, 1.

SWERDLOW, A.J. & GREEN, A. (1987). Melanocytic naevi and melanoma: an epidemiological perspective. *Br. J. Dermatol.*, **17**, 137.